# Pedestrians in Traffic Environments: Ultrafine Particle Respiratory Doses

**DOI:** 10.3390/ijerph14030288

**Published:** 2017-03-09

**Authors:** Maurizio Manigrasso, Claudio Natale, Matteo Vitali, Carmela Protano, Pasquale Avino

**Affiliations:** 1 Department of Technological Innovations, National Institute for Insurance against Accidents at Work, Research Area, via Roberto Ferruzzi 38/40, I-00143 Rome, Italy; claude92@hotmail.it (C.N.); avino@unimol.it (P.A.); 2 Department of Public Health and Infectious Diseases, Sapienza University of Rome, Piazzale Aldo Moro, 5, I-00185 Rome, Italy; matteo.vitali@uniroma1.it (M.V.); carmela.protano@uniroma1.it (C.P.); 3 Department of Agriculture, Environment and Food, University of Molise, via de Sanctis, I-86100 Campobasso, Italy

**Keywords:** ultraFine particles, autovehicular traffic, granulometric size, number concentration, urban air, workday, exposure, dose deposition, human respiratory tract, COPD

## Abstract

Particulate matter has recently received more attention than other pollutants. PM_10_ and PM_2.5_ have been primarily monitored, whereas scientists are focusing their studies on finer granulometric sizes due both to their high number concentration and their high penetration efficiency into the respiratory system. The purpose of this study is to investigate the population exposure to UltraFine Particles (UFP, submicrons in general) in outdoor environments. The particle number doses deposited into the respiratory system have been compared between healthy individuals and persons affected by Chronic Obstructive Pulmonary Disease (COPD). Measurements were performed by means of Dust Track and Nanoscan analyzers. Forty minute walking trails through areas with different traffic densities in downtown Rome have been considered. Furthermore, particle respiratory doses have been estimated for persons waiting at a bus stop, near a traffic light, or along a high-traffic road, as currently occurs in a big city. Large differences have been observed between workdays and weekdays: on workdays, UFP number concentrations are much higher due to the strong contribution of vehicular exhausts. COPD-affected individuals receive greater doses than healthy individuals due to their higher respiratory rate.

## 1. Introduction

Outdoor air pollution is an important health threat for humans in both developed and developing countries. The World Health Organization (WHO) estimated that in 2012 ambient air pollution caused three million premature deaths worldwide. These deaths have been mainly ascribed to exposure to particulate matter (PM) of 10 microns or less in diameter (PM_10_) [1]. The latest scientific research on the association between PM exposure and negative human health outcomes has been focused on fine (PM_2.5_) and UltraFine Particles (UFPs < 100 nm). UFPs play a significant role in PM-induced adverse effects, both for the pulmonary system and other sites such as the cardiovascular and nervous systems [2,3,4,5]. Furthermore, these findings are supported by the inverse relationship occurring between particle size and their toxicity, i.e., particles with smaller granulometric sizes are the most dangerous for human health [6]. In addition, UFPs exhibit some adverse properties such as high surface area to mass ratio, ultrahigh reactivity, and smaller size than the dimensions of cellular structures. These characteristics allow UFPs to easily adsorb organic molecules and reach cellular targets of the pulmonary system and other systems [7,8,9]. In addition, particles with a diameter greater than 2.5 µm are efficiently removed from the atmosphere though dry and wet deposition processes, whereas particles with a diameter less than 1 µm (PM_1_) persist for a longer period, contaminating outdoor and indoor air [10], and can be transported long-range.

Despite all this evidence, UFPs have not yet received the proper attention from environmental policy makers or consideration in air quality regulations. Thus, an important issue in this field is a better understanding of UFP sources, composition, and size distribution, as well as their routes of potential exposure and their impact on adverse outcomes [5]. Regarding the UFP sources, it has been demonstrated that the major contributors to UFP air pollution are anthropogenic emissions, such as combustion engines and power plants [5]. In particular, scientific literature evidences that, in urban areas, UFPs mostly derive from diesel and automobile exhaust [11,12,13]. Thus, it is fundamental to monitor UFP traffic-emissions in order to assess human exposure and to evaluate the related health risks. Difficulties related to this kind of monitoring are the very quick evolution of UFP concentrations and size distribution when they are released into the atmosphere. These phenomena can be influenced by several parameters, such as the meteorological conditions (e.g., wind speed, wind direction, and height of the boundary layer), the levels of some precursor gases (i.e., SO_2_ and NO_x_) and other pre-existing particles [14]. Furthermore, exposure levels to anthropogenic emissions can greatly fluctuate, even at short distances, and are strongly related to personal activities. Consequently, UFP exposure assessment should overcome the limitations of the traditional analyzers installed in fixed monitoring stations [11].

Within this context, the aim of the present study is to estimate the potential UFP exposure of people living in typical urban environments. We performed an intensive monitoring campaign in the urban areas of a big city (Rome, Italy), characterized by different levels of traffic density during the winter season in severe atmospheric conditions (atmospheric stability conditions, high pressure for long periods, pollutant accumulation, traffic restrictions, etc.). Along with PM measurements, we investigated UFP number concentrations and size distributions and evaluated individual exposure. These data were used to assess the doses of particles deposited into the respiratory system for each investigated urban scenario.

This paper represents the first attempt to describe the doses deposited in the respiratory tract of people commuting in a large city. Moreno et al. [15] compared atmospheric contaminants inhaled by travelers during bus, subway train, tram, and walking journeys through the city of Barcelona; this paper was focused on the chemical aerosol speciation, carried out in the 10–300 nm particle range. Many other studies were based on the chemical speciation and the relevant influences on the distribution of transport-related air pollutants in urban air [16,17,18,19,20,21,22,23,24,25,26,27,28], whereas no papers deal with the dose deposited in the human respiratory tract.

## 2. Experiments

Aerosol measurements have been carried out for some typical scenarios currently encountered in urban environments. Measurements have been performed along an urban path in Rome, including areas with different vehicular traffic densities. Furthermore, the ultrafine particle exposure of pedestrians waiting for a bus or standing close to a traffic light along a high traffic density road, waiting to cross the road, was evaluated.

### 2.1. Sampling Sites

**Figure 1:**
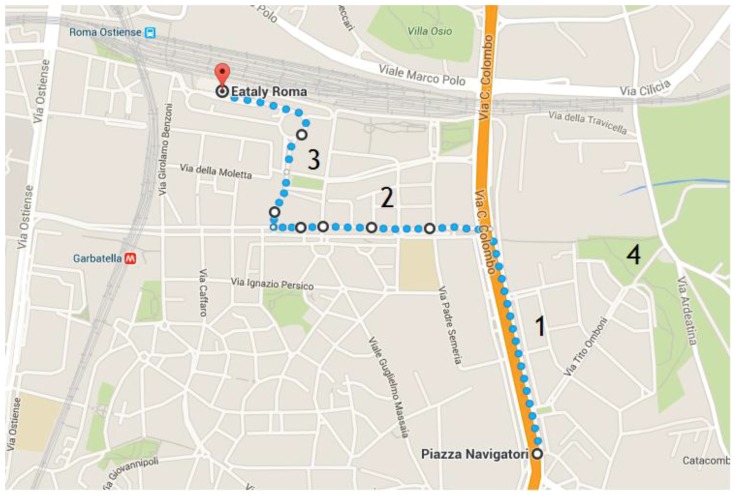
The path from Piazza dei Navigatori to Ostiense train station. Zone 1 = high vehicular traffic density area; zone 2 = medium vehicular traffic density area; zone 3 = low vehicular traffic density area; and zone 4 = Park.

A 40-min walking path was considered, as, on average, this would be the time required to reach a workplace in Rome. The selected path (Figure 1) proceeds from Piazza dei Navigatori to Ostiense train station across a distance of 2.5 km and an estimated time travel of 40–50 min.

The entire sampling campaign was carried out in January–February 2016 for 30 workdays and 6 holidays. During this period, the considered path was followed both on workdays and holidays. The data reported in the present study are representative of a typical daily trend in such a period.

Three different areas can be identified on the selected path (Figure 1): the first one, labeled “1”, is along “Via Cristoforo Colombo” and is characterized by high vehicular traffic density (cars, buses, trucks, motorcycles); the second one, labeled “2”, runs along “Circonvallazione Ostiense Street” and is at medium traffic density; and the last one, labeled “3”, “Piazza 12 Ottobre 1492”, is a low vehicular traffic density area.

By using a camera, the number of vehicles circulating per unit of time was evaluated; on average, about 200 vehicles (e.g., cars, buses, and motorcycles) per minute passed in zone 1, an average of 44 vehicles per minute passed in zone 2, and 2 vehicles per minute passed in zone 3 on workdays and 10 cars on holidays.

It is important to point out that, during the sampling period, the weather in Rome was unfavorable to the pollutant dispersion. High barometric pressure characterized the entire campaign period and induced the Rome City Mayor to limit the car circulation on workdays and to prohibit it on holidays. The data reported as holidays refer to Sundays, when car traffic was completely halted, except emergency vehicles, buses, and taxis.

Aerosol measurements were performed to evaluate the exposure of pedestrians waiting at a bus stop and at a traffic light close to a high traffic density road. In the first case, an average waiting time of about 20 min was considered. In the second case, 50 s were considered on workdays and 40 s on holidays (the duration of the red traffic light). Both operations were carried out on workdays and on holidays.

Throughout the campaigns, a baseline measurement was performed in order to evaluate indirect vehicular traffic influence; the measurements were carried out in Villa Ada Park, a park considered the background area of Rome in terms of air pollution and which is quite far from direct vehicular traffic emissions.

### 2.2. Measurement Equipment

Different PM size fractions have been investigated along with the temporal trend of particle number concentration in the 11–365 nm size range. A backpack was equipped with a portable DustTrack system and a Nanoscan instrument. The DustTrakTM II Aerosol Monitor 8532 (TSI, Shoreview, MN, USA), a handheld battery-operated, data-logging, light-scattering laser photometer, simultaneously measures both mass and size fractions (PM_1_, PM_2.5_, PM_4_, or respirable fraction, PM_10_, and Total PM size fractions). It uses a sheath air system that isolates the aerosol in the optics chamber to keep the optics clean for improved reliability and low maintenance. A NanoScan SMPS 3910 (TSI), which adopts a scanning mobility particle sizing technology, was also used to measure particle number concentrations in the range 10–365 nm [29]. Aerosol concentrations were measured with 60 s time resolutions in thirteen size channels (i.e., 11.5 nm, 15.4 nm, 20.5 nm, 27.4 nm, 36.5 nm, 48.7 nm, 64.9 nm, 86.6 nm, 115.5 nm, 154.0 nm, 205.4 nm, 273.8 nm, and 365.2 nm). The attention was focused on ultrafine particles, i.e., on size channels from 11.5 nm to 115.5 nm (and the first three modes, which are important in the accumulation mode).

Two probes (anti-electrostatic tubes) were placed on the jacket lapel to get detailed information on personal exposures, on opposite sides in order to avoid interferences and to prevent air vortices that could affect the measurement.

### 2.3. Dosimetry Calculations

The methodology of particle dose calculation has been thoroughly described in previous papers [30,31,32,33,34,35,36], where the particle deposition in the human respiratory system was evaluated using the Multiple-Path Particle Dosimetry model (MPPD v2.1, ARA 2009, ARA, Arlington, VA, USA). This model calculates, in the respiratory tract, the deposition and clearance of mono- and poly-disperse aerosols in the range from ultrafine to coarse particles in the respiratory tract [37,38]. A stochastic lung model was considered because it provides more realistic lung geometry than the symmetric one considered in the International Commission on Radiological Protection (ICRP) model [39]. In the MPPD model, the ten stochastic lungs are ordered according to size (total number of airways) from the smallest to the largest, and the approximate size percentile of each lung is provided [40]; the 60th percentile human stochastic lung has been considered in this study. The following settings were considered in the MPPD model: (i) an uniform expansion of the lung; (ii) an upright body orientation; and (iii) nasal breathing with an inspiratory fraction and a no pause fraction. Moreover, the following parameters were used for a Caucasian adult male under the sitting-awake condition based on the ICRP report [39]: (i) a functional residual capacity (FRC) of 3300 mL; (ii) an upper respiratory tract (URT) volume equal to 50 mL; (iii) a 10.7-min^−1^ and 13.1-min^−1^ breathing frequency for healthy and COPD (Chronic Obstructive Pulmonary Disease) patients, respectively [41]; and (iv) an air volume inhaled during a single breath (tidal volume, *V_t_*) of 1.25 L [42].

The total doses of particles deposited in the human respiratory tract vs. time has been estimated for healthy individuals (*D^Healthy^*) and individuals affected by Chronic Obstructive Pulmonary Disease (COPD) [43] according to the following equations:

$$D^{Healthy}\left(t\right)=\sum_{i=1}^{13}D_{i}^{Healthy}\left(t\right)\;\mathrm{with}\;D_{i}^{Healthy}\left(t\right)=F_{i}^{Healthy}\times C_{i}\left(t\right)\times V_{t}$$

$$D^{COPD}\left(t\right)=\sum_{i=1}^{13}D_{i}^{COPD}\left(t\right)\;\mathrm{with}\;D_{i}^{COPD}\left(t\right)=F_{i}^{COPD}\times C_{i}\left(t\right)\times V_{t}$$

where *F_i_^Healthy^* and *F_i_^COPD^* are the fractions of the particle deposited doses in the respiratory tract of healthy people and individuals affected by COPD, respectively; *C_i_(t)* is the particle number concentration measured by NanoScan; and *V_t_* is the tidal volume, i.e., the volume of air involved in each respiratory act. The *F_i_^Healthy^* and *F_i_^COPD^* deposition fractions were derived from Löndahl et al. [41]; the authors measured experimentally the deposition fractions of aerosol emitted from diesel engines on a group of eight healthy volunteers and 10 volunteers suffering from COPD. A *V_t_* of 0.86 L has been assumed for both cases, whereas a respiratory frequency of 10.7 min^−1^ and 13.1 min^−1^ has been assumed for healthy people and COPD-affected people, respectively.

## 3. Results

### 3.1. Aerosol Number Size Distributions

#### City Path Analysis

The methodology reported in the Experimental section was applied to the overall campaign. First, the different fractions of aerosol were estimated; Table 1 shows the data on PM concentrations measured during the workdays.

The comparison between the fraction levels evidenced that PM_2.5_ represented 95% of PM_10_ and that 94% of PM_10_ was made of PM_1_.

A similar situation occurred for the measurements performed during holidays (Table 2), wherein PM_1_ (average 32 µg·m^−3^) represented 91% of the PM_10_ (average 35 µg·m^−3^).

**Figure 2:**
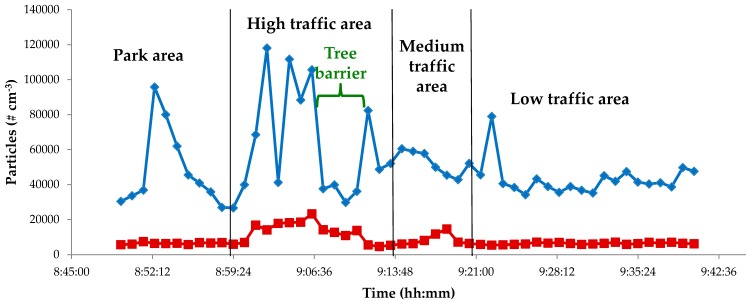
Typical trend of the submicron particle concentration values (# cm^−3^) sampled along an urban path during workdays (blue line) and holidays (red line).

Table 3 shows the Pearson’s correlations among particulate matter at different granulometric sizes. Correlation coefficients were higher for fractions from PM_1_ to PM_10_; they ranged from 0.999 for PM_2.5_–PM_1_ to above 0.9 for other fractions below PM_10_. These PM fractions were so highly correlated because, in the investigated periods, PM pollution was mainly due to fine aerosol deriving from anthropogenic sources. Correlation coefficients between total PM and the other PM fractions were lower (0.66–0.74), very likely because of the resuspension of road dust due to the nearby vehicular traffic. The PM composition of Rome’s urban air, extensively studied by Perrino et al. [44] and Avino et al. [45], was characterized by a crustal contribution that ranged between 7%–30%, whereas the remaining amount was due to anthropogenic sources. This last fraction was formed by 30%–40% of carbonaceous particulate matter [46] and 20%–35% of ammonium sulphate and nitrate. Minor percentage components, but ones that were really important for their public health relevance, included Polyciclic Aromatic Hydrocarbons (PAHs), nitro-PAHs, oxo-PAHs [47], and heavy metals [48]. Specifically, PM_1_ was the most significant component of PM_10_ and, therefore, it is important to analyze its size distribution. To this aim, a NanoScan instrument was used. Figure 2 shows the total concentration values in two typical days, a workday and a holiday, along the considered urban path; the profiles represent the average of all the measurements performed during the entire campaign.

The first relevant finding is that during workdays the submicron particle concentration reached a value of about 120,000 # cm^−3^ and it never dropped below 25,000 # cm^−3^. However, when the vehicular traffic was completely halted (no vehicles passing-by due to red traffic light), data were very different; the submicron particle concentrations were considerably lower (about 23,000 # cm^−3^), and the minimum result was approximately 5000 # cm^−3^. As reported in Figure 2, the highest concentrations were reached along Via Cristoforo Colombo (10 car lanes, track width 25 m), a high traffic density road during workdays.

The area tagged “Park area” (Figure 2) is located close to a high traffic density road; the peak value detected was due to truck emissions because of work in progress. Although truck emissions inside a park are not a currently encountered situation, it is not so rare when the park is close to an avenue with 10 lanes and frequent maintenance works. Therefore, this data set can be considered as representative of a real exposure scenario in a densely populated area of Rome. In the area tagged “High traffic area” (along Via Cristoforo Colombo), the highest values were due to the continuous transit of many vehicles during the “green” traffic light, while the lowest level occurred when the traffic light was red. Moving on the path, the particle concentration levels drop, due to the presence of trees, which act as a natural barrier to the particles and allow a reduction of the exposure level. The last peak in the “High traffic area” was due to vehicles waiting to cross the road at the traffic light. In the area tagged as “Medium traffic area”, particle concentrations were almost constant and were lower than in the previous zone. The same occurred in the “Low traffic area” zone, where the only (very low) peak was recorded near a market with several vehicles nearby. During holidays, the submicron particle concentration values were very low (below 20,000 # cm^−3^) and almost constant over the time.

Table 4 summarizes the aerosol size distribution data measured in both workdays and holidays. The Coefficient of Variation (CV %) gives an index of the variability of the measurements.

During workdays, the particle concentrations for size channels from 11.5 nm to 48.7 nm showed high CVs (up to 100% for the first three channels) due to the fast temporal evolution of such particles, whereas the CVs were below 50% for size fractions above 48.7 nm. During holidays, the trend was quite similar, although with lower values. All these data confirm that the different particle behavior strictly depends on the granulometric size fractions; nucleation particles are quickly generated by autovehicular exhausts, and their concentration rapidly drops due to diffusion, coagulation, and deposition mechanisms.

UFP percentage contribution was about 75% and confirms previous measurements, performed in a street canyon in downtown Rome, in which their percentage ranged between 70%–95% [49,50]; studies performed in other urban environments, in cities other than Rome, reported a percentage contribution of particles below 100 nm of about 80% of the total particle number concentration [51,52,53,54,55].

**Figure 3:**
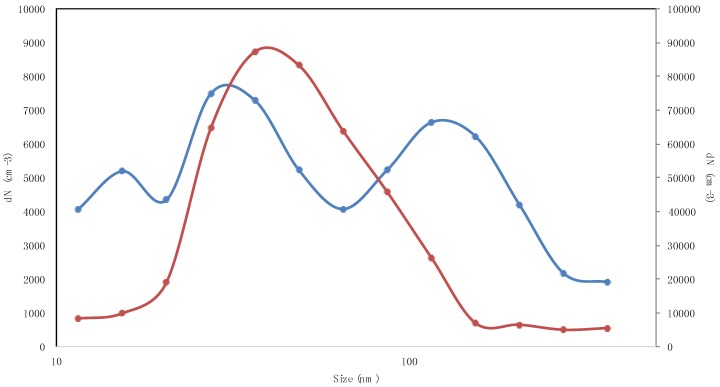
Comparison of the aerosol size distribution profiles in the “waiting for” (blue line) and “bus coming” (red line) scenarios.

The Pearson’s correlation coefficients among different size fractions (Table 5) evidence the common origin of ultrafine particles. For instance, in Table 5, coefficient correlations above 0.7 up to 0.98, calculated for particles ranging from 36 nm to 86 nm, denote the contribution of diesel engine emissions. This occurrence is confirmed by data shown in Figure 3, in which the size distribution for a diesel engine bus (“waiting for” scenario) is reported.

**Figure 4:**
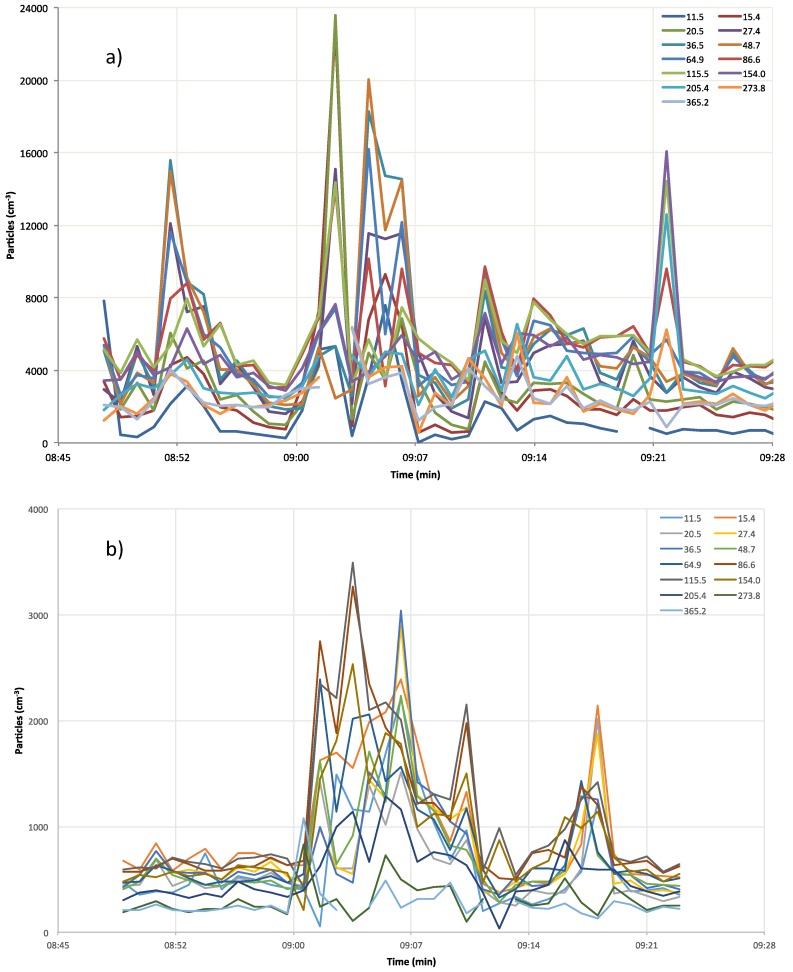
Typical trends of different size fractions during workdays (**a**) and holidays (**b**).

The temporal concentration levels of each NanoScan size channel are reported in Figure 4. Peak concentrations are due to nucleation particles (NanoScan size channel from 11.5 nm to 48.7 nm) that were continuously released by the intense autovehicular emissions. In contrast, the greater contribution in the low traffic zone was due to larger particles because nucleation particles were rapidly dispersed and removed through coagulation and deposition mechanisms. These features have an important influence on the doses deposited into the human respiratory tract. This issue will be discussed in the Section 3.2, both for healthy people and patients affected by COPD.

People waiting at bus stops is a common scenario in urban areas. In such a simulated scenario, PM_10_ (overall mean value 154 µg·m^−3^) was composed of 94% PM_1_ (overall mean value 144 µg·m^−3^) (data not shown), and the exposure to submicron particles is very high on workdays due to long bus-waiting times and high particle concentration. Total particle concentrations between 50,000 and 100,000 # cm^−3^ and peak concentrations as high 415,000 # cm^−3^ were measured on bus arrivals.

Figure 3 shows the aerosol number size distributions representative of the “waiting” and “bus coming” scenarios. In the first case, a trimodal size distribution was measured (with modes at about 15.4 nm, 27.4 nm, and 115.5 nm). In the second case, a single mode of 36.5 nm was measured. In particular, the “bus coming” scenario is characterized by a monomodal distribution, centered at about 36.5 nm, coherent with the measurements performed by Kittelson for diesel engine exhaust size distribution [56]. The differences between the two size distributions reported in Figure 3 are due to the different bus engine loads [57] occurring in the “waiting” and “bus coming” scenarios.

Similar trimodal and monomodal distributions were measured upon waiting at traffic lights. Bimodal size distributions (modes at 15.4 nm and 115 nm) were also measured, depending on the engine emissions and loads. Total particle number concentrations as high as 100,000 # cm^−3^ were measured for traffic lights in red status [58] due to the simultaneous presence of a high number of vehicles waiting in the queue, whereas, during the green traffic light, particle levels below 40,000 # cm^−3^ were measured.

### 3.2. Dose Deposited in the Human Respiratory Tract

Table 6 summarizes the main findings in terms of particle doses for healthy and COPD-affected people, respectively, along with the dose increment % estimated for COPD-affected individuals.

**Figure 5:**
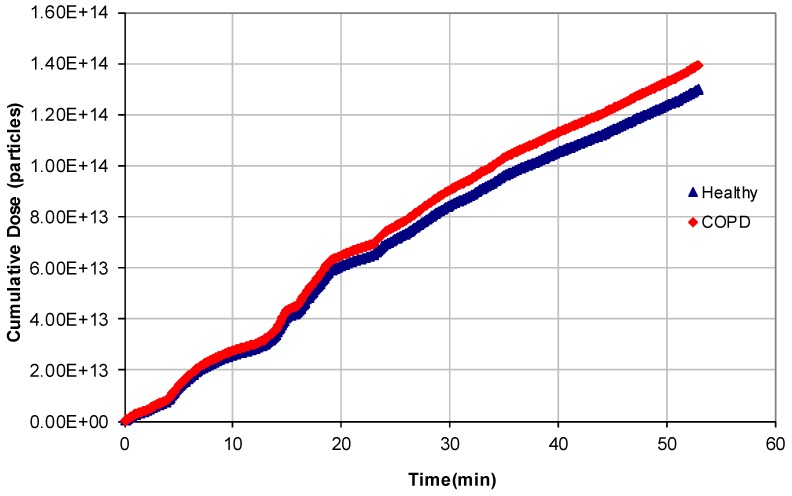
Typical profile of the cumulative doses of particles deposited in the human respiratory system of healthy persons and patients affected by Chronic Obstructive Pulmonary Disease (COPD) along a city path on workdays.

**Figure 6:**
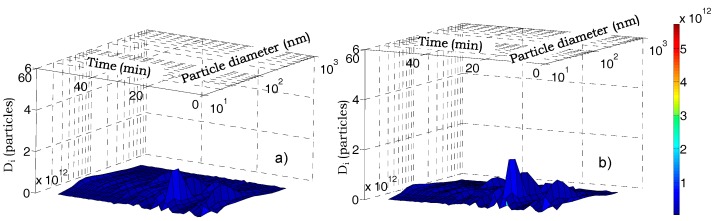
Particle number doses deposited in 1 min time intervals in the human respiratory system of healthy (**a**) and COPD-affected (**b**) persons as functions of particle sizes and time along a city path in workdays.

Figure 5 shows the cumulative doses of particles deposited in the human respiratory system during the city path (workdays) by comparing healthy persons and patients affected by COPD, whereas Figure 6 shows the particle doses deposited in the respiratory system of healthy and COPD-affected persons in 1 min time intervals (the Nanoscan scan time) as functions of time and of particle diameter. People with COPD disease received higher particle doses than healthy persons, due to their increased respiratory rate, i.e., the difference is about 7.3% (city path). Similar trends were found for holiday dosimetry estimates; in this case, the difference between the two profiles was 7.7%, whereas the particle number deposited was about five orders of magnitude less that calculated for the previous simulation (Table 6).

**Figure 7:**
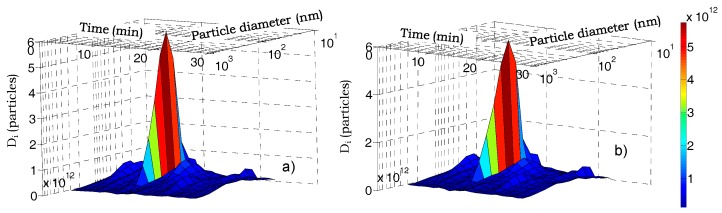
Particle number doses deposited in 1 min time intervals in the human respiratory system of healthy (**a**) and COPD-affected (**b**) persons as functions of particle sizes and time for the bus stop waiting scenario on workdays.

For the “bus waiting” scenario, the cumulative dose for COPD-affected individuals was about 5.2% and 8.4% greater than for healthy individuals during workdays and holidays, respectively. Figure 7 shows the particle doses deposited into the respiratory system of healthy and COPD-affected persons in 1 min time intervals as functions of time and of particle diameter for the “bus waiting” scenario. In comparison with the city walk path, peak doses were about 3-fold higher and in the same range from 20.5 nm to 36.5 nm as for the city walk path. However, the peak doses for the “bus waiting” scenario were about threefold higher for healthy individuals than for COPD-affected individuals.

**Figure 8:**
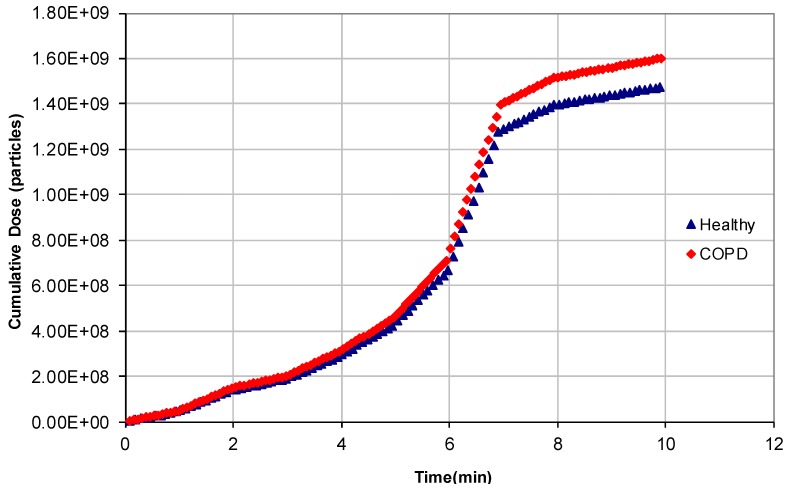
Cumulative particle doses deposited in the human respiratory system of healthy people and persons affected by COPDs; the simulation was performed while the pedestrian waited for green light at a traffic light on holidays.

Finally, simulations of individuals waiting at a traffic light were also performed. Figure 8 shows the cumulative particle doses deposited into the human respiratory system of healthy and of COPD-affected individuals waiting at a traffic light on a typical workday. As observed in the previous scenario, cumulative particle doses were 7.71% higher for persons suffering from COPD than for healthy persons during workdays and >8.54% during holidays.

It should be considered that waiting for the “green” traffic light for ten minutes in most cases is an unlikely situation; nonetheless, in some other cases, such as a police officer standing outside near a traffic light for much more than ten minutes, the simulation is certainly much more significant.

## 4. Conclusions

This paper deals with an important potential threat to human health, ultrafine particle exposure and the relevant dose deposited into the respiratory tract of pedestrians in big urban areas such as Rome, considering both healthy people and more susceptible individuals, such as those affected by COPD. UFPs are becoming an issue investigated by several authors, and data sets are being collected in different urban areas. However, health effects have been mainly associated with particulate matter (i.e., PM_10_ and PM_2.5_) [59], whereas studies that correlate UFPs and human health in outdoor environments are scarce. Moreover, health effects are not directly linked to particle concentration, but they are associated with the particle doses deposited into the respiratory system. To date, dosimetry data are not so widely reported in literature for urban environments. To the authors’ knowledge, this is the first paper that investigates the deposition dose for healthy and COPD-affected individuals in real urban scenarios. This kind of evaluation is highly relevant because, in 2013, COPD affected 329 million people or nearly five percent of the global population (males and females with the same incidence) [60,61] with consequently very high social costs (i.e., $2.1 trillion in 2010 [62]). The present study shows that individuals with COPD receive higher particle doses (+7%–9%) compared to healthy people. The measurements performed in downtown Rome display great differences between workdays and holidays, with the former reaching submicron particle values definitely higher than the latter and thus highlighting the strong contribution of autovehicular traffic to ultrafine particle generation.

## Figures and Tables

**Table 1:** Particulate matter (PM) data (as µg·m^−3^) measured using DustTrack during the simulation of the city path during workdays. Variability is calculated as Coefficient of Variation (CV %).

Parameter	PM_1_	PM_2.5_	PM_4_	PM_10_	Total PM
**Mean**	129	130	131	137	155
**Median**	125	126	127	132	138
**Min**	98	98	99	100	100
**Max**	316	324	341	412	1160
**60%**	128	129	130	135	145
**80%**	136	137	138	145	169
**95%**	154	155	157	168	239
**St. Dev.**	16	17	18	24	59
**Variability**	12	13	14	18	38

**Table 2:** PM data (as µg·m^−3^) measured using DustTrack during the simulation of the city path during holidays (variability calculated as CV %).

Parameter	PM_1_	PM_2.5_	PM_4_	PM_10_	Total PM
**Mean**	32	32	33	35	45
**Median**	30	30	30	31	32
**Min**	20	20	20	20	20
**Max**	251	252	252	283	1600
**60%**	31	31	32	33	34
**80%**	34	34	35	37	45
**95%**	46	46	47	53	105
**St. Dev.**	11	11	12	17	50
**Variability**	34	34	36	47	112

**Table 3:** Pearson’s correlation between different PM sizes during workdays (**a**) and holidays (**b**).

**PM_1_**	**PM_2.5_**	**PM_4_**	**PM_10_**	**Total PM**	**(a)**
1	0.999	0.995	0.934	0.660	PM_1_
	1	0.997	0.943	0.668	PM_2.5_
		1	0.959	0.686	PM_4_
			1	0.740	PM_10_
				1	Total PM
**PM_1_**	**PM_2.5_**	**PM_4_**	**PM_10_**	**Total PM**	**(b)**
1	0.999	0.994	0.913	0.686	PM_1_
	1	0.996	0.922	0.692	PM_2.5_
		1	0.946	0.705	PM_4_
			1	0.740	PM_10_
				1	Total PM

**Table 4:** Particle measurements (# cm^−3^) performed by Nanoscan during the city path on workdays (**a**) and on holidays (**b**) (variability calculated as CV %).

**Parameter**	**11.5**	**15.4**	**20.5**	**27.4**	**36.5**	**48.7**	**64.9**	**86.6**	**115.5**	**154.0**	**205.4**	**273.8**	**365.2**
**(a)**													
Mean	1344	2641	2958	4480	5083	5097	5172	5566	5531	4699	3538	2651	2464
Median	635	1655	2215	3403	3991	4090	4768	4950	5041	4329	3082	2253	2166
Min	31	567	759	1371	1829	2088	2535	3031	3187	2862	1817	568	852
Max	7818	22,820	23,579	15,113	18,271	20,021	16,193	14,068	14,399	16,091	12,613	6360	6302
60%	691	1816	2392	3674	4505	4593	4988	5296	5347	4388	3223	2363	2286
80%	1890	3110	3449	5422	5814	5621	5955	6543	6000	5195	3991	3510	3039
95%	5221	6998	5466	11,545	14,619	12,827	10,256	9632	8385	6650	5033	5316	4072
St. Dev.	1729	3359	3182	2915	3473	3406	2511	2121	2143	1877	1560	1183	906
Variability	129	127	108	65	68	67	49	38	39	40	44	45	37
**Parameter**	**11.5**	**15.4**	**20.5**	**27.4**	**36.5**	**48.7**	**64.9**	**86.6**	**115.5**	**154.0**	**205.4**	**273.8**	**365.2**
**(b)**													
Mean	661	952	629	770	765	730	826	1044	1103	888	542	306	278
Median	446	697	472	562	551	500	582	674	713	611	412	253	232
Min	54	378	250	372	354	281	329	496	426	206	36	100	131
Max	2210	2386	2023	2890	3035	2231	2384	3271	3494	2531	1287	827	1080
60%	508	765	531	601	574	569	602	727	882	775	521	290	250
80%	1056	1567	732	1092	1084	963	1165	1453	1538	1193	679	410	298
95%	1551	2098	1447	1568	1448	1648	2029	2465	2253	1831	1150	587	479
St. Dev.	484	575	401	517	521	449	523	699	711	520	274	156	163
Variability	73	60	64	67	68	62	63	67	64	59	51	51	59

**Table 5:** Pearson’s correlation coefficient among the submicron particles in different size fractions on workdays (**a**) and on holidays (**b**). Values > 0.7 are reported in italic.

11.5	15.4	20.5	27.4	36.5	48.7	64.9	86.6	115.5	154.0	205.4	273.8	365.2	
													**(a)**
1	*0.707*	0.590	0.608	0.561	0.490	0.488	0.434	0.285	0.131	0.091	0.161	0.265	11.5
	1	*0.932*	*0.793*	0.437	0.322	0.479	*0.703*	0.598	0.283	0.233	0.310	0.355	15.4
		1	*0.773*	0.333	0.216	0.431	*0.751*	0.661	0.289	0.157	0.146	0.205	20.5
			1	*0.842*	*0.741*	*0.783*	*0.706*	0.461	0.213	0.139	0.203	0.288	27.4
				1	*0.976*	*0.889*	0.492	0.157	0.077	0.136	0.253	0.347	36.5
					1	*0.927*	0.486	0.124	0.068	0.151	0.277	0.360	48.7
						1	*0.761*	0.417	0.252	0.221	0.269	0.287	64.9
							1	*0.872*	0.596	0.424	0.228	0.040	86.6
								1	*0.861*	*0.717*	0.294	−0.202	115.5
									1	*0.909*	0.508	−0.125	154.0
										1	*0.806*	0.213	205.4
											1	*0.727*	273.8
												1	365.2
													**(b)**
11.5	15.4	20.5	27.4	36.5	48.7	64.9	86.6	115.5	154.0	205.4	273.8	365.2	
1	*0.867*	0.613	*0.787*	*0.765*	0.654	0.509	0.547	0.631	*0.719*	*0.790*	0.276	−0.063	11.5
	1	*0.883*	*0.836*	*0.777*	*0.795*	*0.765*	*0.765*	*0.777*	*0.787*	*0.760*	0.152	−0.050	15.4
		1	*0.857*	*0.748*	*0.731*	0.653	0.602	0.576	0.550	0.491	0.026	−0.041	20.5
			1	*0.964*	*0.826*	0.549	0.475	0.498	0.544	0.591	0.202	−0.022	27.4
				1	*0.902*	0.583	0.457	0.464	0.519	0.609	0.285	0.008	36.5
					1	*0.850*	*0.703*	0.651	0.646	0.666	0.226	0.009	48.7
						1	*0.951*	*0.881*	*0.810*	0.686	0.027	−0.060	64.9
							1	*0.973*	*0.907*	*0.732*	−0.051	−0.123	86.6
								1	*0.974*	*0.786*	−0.044	−0.192	115.5
									1	*0.867*	0.047	−0.215	154.0
										1	0.399	0.005	205.4
											1	*0.819*	273.8
												1	365.2

**Table 6:** Total doses of aerosol deposited in the human respiratory system, relevant UltraFine Particle (UFP) % contribution for the healthy and COPD-affected individuals, and % dose increments estimated for COPD-affected individuals in comparison with healthy individuals (Δ %), both on workdays and holidays.

Scenario	Exposure Time (min)	UFPs/Total (%)	Total Dose (Particle)	Δ (%)
*workdays*				
city path	52/52	87.6/85.8	1.30 × 10^14^/1.40 × 10^14^	+7.3
w traffic light	9/9	92.1/90.8	3.56 × 10^13^/3.84 × 10^13^	+7.7
w bus stop	22/22	92.1/91.0	1.04 × 10^14^/1.09 × 10^14^	+5.2
*holidays*				
city path	34/34	90.3/89.0	1.72 × 10^9^/1.85 × 10^9^	+7.7
w traffic light	9/9	94.3/93.6	1.47 × 10^9^/1.60 × 10^9^	+8.5
w bus stop	22/22	88.5/87.0	1.14 × 10^9^/1.24 × 10^9^	+8.4
